# Seasonal Variation of Tropical Savanna Altered Agronomic Adaptation of Stock-6-Derived Inducer Lines

**DOI:** 10.3390/plants11212902

**Published:** 2022-10-28

**Authors:** Paepan Sintanaparadee, Abil Dermail, Thomas Lübberstedt, Kamol Lertrat, Sompong Chankaew, Vinitchan Ruanjaichon, Nittaya Phakamas, Khundej Suriharn

**Affiliations:** 1 Department of Agronomy, Faculty of Agriculture, Khon Kaen University, Khon Kaen 40002, Thailand; 2 Department of Agronomy, Iowa State University, Ames, IA 50011, USA; 3 Plant Breeding Research Center for Sustainable Agriculture, Faculty of Agriculture, Khon Kaen University, Khon Kaen 40002, Thailand; 4 National Center for Genetic Engineering and Biotechnology (BIOTEC), 113 Thailand Science Park, Pahonyothin Road, Khlong Nueng, Khlong Luang, Pathum Thani 12120, Thailand; 5 Department of Plant Production Technology, School of Agricultural Technology, King Mongkut’s Institute of Technology Ladkrabang, Bangkok 10520, Thailand

**Keywords:** haploid production, inducer maintenance, *R1-nj* biomarker, seed set, *Zea mays* L.

## Abstract

Tropicalization is one of the major objectives in breeding haploid inducers to address the poor adaptation of temperate haploid inducers in doubled haploid production in tropical maize. Gaining a better understanding of weather profiles in targeted agroecology is important. This study aimed to investigate the seasonal variation of tropical savanna climate and its impact on agronomic traits and haploid induction rate (HIR) of Stock-6-derived haploid inducer lines. A total of 14 haploid inducers were evaluated across two typical growing seasons between 2020 and 2021. Weather data were collected on daily minimum and maximum temperatures, relative humidity, precipitation, and solar radiation whereas phenotypic data were recorded on plant phenology, tassel attributes, plant stature, ear components, inducer seed rate (ISR), and HIR. The effects of season, genotype, and genotype by season were significant for all traits except season factor on ISR. Seasonal variation existed where the dry season was more suitable for haploid induction and inducer maintenance, as haploid inducers revealed better agronomic performance and seed set, delayed flowering dates, and higher HIR. Since the crossover performance of haploid inducers over seasons was detected, further implications on genotype selection in each season are discussed.

## 1. Introduction

Doubled haploid (DH) technology has become a part of worldwide commercial maize breeding programs since it fastens the formation of homozygous inbred lines from 6–8 generations to only 2 generations [1]. Haploid induction, the first step of DH technology, may be accomplished with two systems: in vivo and in vitro methods [2]. In vivo haploid induction is preferable and widely adopted in maize [1]. Through this system, haploid inducers act as pollinators of source (donor) germplasm to produce maternal haploids [1]. The proportion between the number of induced haploids and total seeds per induction cross is called haploid induction rate (HIR). The first haploid inducer was Stock 6 with 2.3% of HIR [3], and more recent haploid inducers have HIRs ranging from 8–15% [4,5,6,7,8,9,10]. Most modern haploid inducers are adapted to temperate regions. When introduced to the tropics, poor tropical adaptation results in shorter plant stature, poor flowering synchrony, pollen production, and seed set, and susceptibility to major tropical diseases [11]. This maladaptation syndrome complicates haploid induction and inducer maintenance, hindering the rapid adoptions of temperate inducers in tropical maize breeding programs [12]. Thus, haploid inducers with high HIR and tropical adaptation are essential for use in tropical regions. The International Maize and Wheat Improvement Center (CIMMYT) in collaboration with the University of Hohenheim developed the first generation of tropically adapted inducer lines (TAILs) [11], and recently released CIM2GTAILs as the second generation of TAILs possessing 8–15% of HIR and good adaptation to both tropical and subtropical environments [13]. These inducers are available for a licensing fee; however, the cost can be unaffordable for seed start-ups.

Thailand has a typical tropical savanna climate [14] that covers three seasons, namely, rainy (May to October), cool dry (October–February), and summer dry (February–May). This climate has several features as follows: 1200–1400 mm of rainfall per year, 63–84% of relative humidity, 13–24 °C of seasonal minimum temperature, and 30–40 °C of seasonal maximum temperature (Thai Meteorological Department, Thailand. Available online: http://www.tmd.go.th (accessed on 8 May 2022)). Temperature is one of the critical parameters determining the adaptation and HIR of haploid inducers. Kebede et al. [15] reported that the winter season was more suitable for haploid induction than the summer season in subtropical Mexico. In contrast, De La Fuente et al. [2] noticed higher HIR in a warmer than in a cooler summer under a temperate climate in Iowa. However, there is no study reporting the weather variability and its effect on the agronomic adaptation and HIR of maize haploid inducers under tropical savanna climates.

Khon Kaen University in Thailand has initiated breeding haploid inducers by introducing the temperate inducer ‘Stock 6’, which is a public maize haploid inducer, to Thai maize germplasm possessing tropical backgrounds, aiming to develop new haploid inducers showing agronomic adaptation for tropical savanna regions and sufficient HIR [16]. Favorable traits for tropical maize and vegetable corn include earliness, shorter plant stature, and high grain yield [17,18]. In addition to those criteria, maize haploid inducers with large tassel size, longer pollen-shed duration, high pollen production, and resistance to ear rot are preferable to optimize self- and cross-pollinations for haploid seed production in induction nurseries and inducer maintenance, respectively [19]. Therefore, this study aimed to investigate the effect of seasonal variation of tropical savanna climate on agronomic traits and the HIR of Stock-6-derived haploid inducer lines. Information obtained in this study will assist breeders by means of screening their haploid inducers against different seasons of haploid induction to achieve optimal haploid production and inducer maintenance.

## 2. Results

### 2.1. Analysis of Variance

Season (S) effect was highly significant on all measured traits except on inducer seed rate (ISR) (Table 1). The effects of genotype (G) and the interaction between season and genotype (GSI) were highly significant on all observed traits. Genotype accounted for the highest proportion of source of variance on ISR, ear length (EL), husk cover (HC), GR, plant height (PH), ear height (EH), primary tassel branch (PTB), and total tassel branch (TTB). Season largely contributed to ear weight (EW), ear diameter (ED), row number per ear (NRE), seed number per row (NSR), cob diameter (CD), and seed diameter (SD). The GSI was predominant for haploid induction rate (HIR).

The significance of genotype indicated the phenotypic variability among 14 haploid inducer lines on overall agronomic performance, yield components, and HIR. The significance of season suggested that phenotypic means of inducers on most traits changed in different growing seasons. The predominance of the GSI on HIR implied that the HIR of inducer lines was sensitive to seasonal variation, and each genotype expressed different responses to different growing seasons.

### 2.2. Seasonal Variation and Plant Phenology

**Figure 1:**
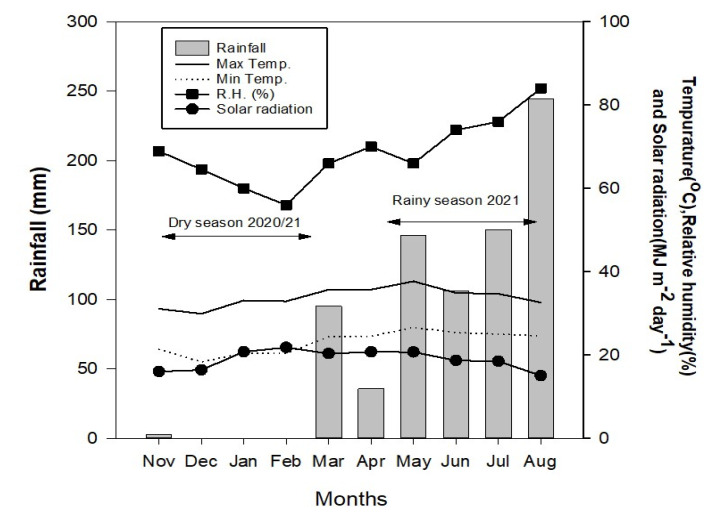
Total rainfall, daily maximum and minimum temperatures, relative humidity, and solar radiation during the experiment in the dry season of 2020/2021 and the rainy season of 2021.

The daily minimum, average, and maximum temperatures during the dry season were 18.3 °C, 26.0 °C, and 33.1 °C, respectively, which were lower than those during the rainy season which had the daily minimum, average, and maximum temperatures of 24.5 °C, 30.2 °C, and 37.7 °C, respectively (Figure 1). Total precipitation in the dry season was much lower than that in the rainy season, as indicated by 2.6 mm against 682.5 mm. Relative humidity during the dry season (56 to 69%) was lower than that during the rainy season (66 to 84%). Meanwhile, the solar radiation of the dry season (21.8 MJ m^−2^ day^−1^) was slightly higher than that of the rainy season (20.7 MJ m^−2^ day^−1^). These results indicated that seasonal variation between the dry and rainy seasons existed.

**Figure 2:**
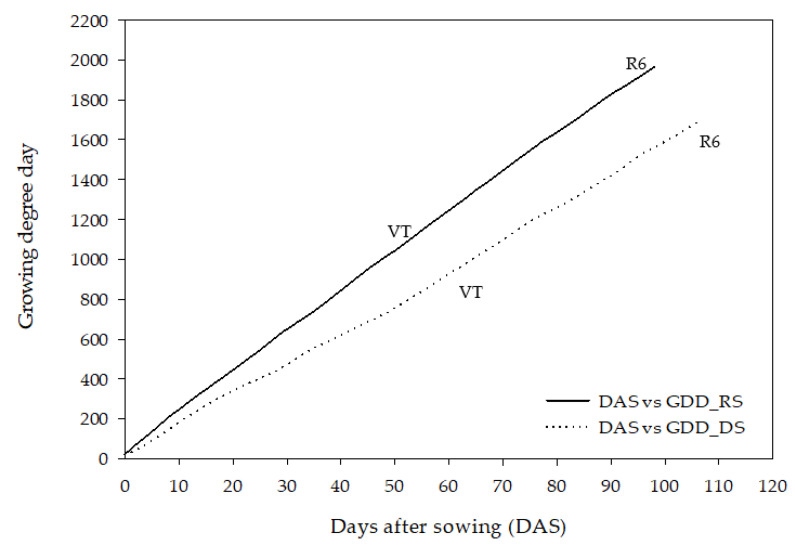
Phenology of overall haploid inducer lines evaluated in the rainy season (RS) and the dry season (DS) expressed in days after sowing and GDD.

The phenology was expressed in days after sowing (DAS) and growing degree days (GDD) (Figure 2). Haploid inducers grown in the rainy season required 1104.9 of GDD or 53 days to anthesis (VT stage) while the same set of genotypes in the dry season required 945.2 of GDD or 61 DAS. In regard to achieving physiological maturity (R6 stage), haploid inducers in the rainy season required 1965.4 of GDD or 98 DAS while it required at least 1689.3 of GDD or 106 DAS. This result indicated that the phenology of overall haploid inducer lines grown in the rainy season was faster than in the dry season, as indicated by higher GDDs but shorter days to anthesis and maturity.

### 2.3. Performance of Maize Haploid Inducers against Seasonal Variation

Maize haploid inducers grown in the dry season showed a slightly lower germination rate than in the rainy season (Table 2). On the contrary, these inducers had significantly higher values of PSD, DTA, DSI, PH, EH, PTB, TTB, TSL, and SPL in the dry season than in the rainy season, indicating that the weather profiles of the dry season conditioned maize inducers to delay the flowering dates, extend the duration of pollen shed, broaden the tassel architecture, and enhance the vegetative growth that resulted in higher plant stature. Likewise, the considerable increases in genotypic means in EW, ED, NRE, NKR, CD, and KD were noticed in the dry season while slight increases in the means were found in EL and HC. These results illustrated that the dry season may provide suitable conditions for ear development and seed set of maize inducers. Two major parameters, HIR and ISR, showed higher values in the dry season than in the rainy season, suggesting that both activities of haploid induction and inducer seed maintenance were more efficient in the dry season.

### 2.4. Interaction between Genotype and Season in Maize Haploid Inducers

In the dry season, KHI 65 and KHI 66 had the highest HIR (3.4% and 3.3%, respectively) among 14 haploid inducers, followed by KHI 54 (2.5%) (Table 3). In regard to ISR, the highest values were noticed in four haploid inducers, namely, KHI 47 (94.2%), KHI 42 (93.6%), KHI 5 (92.0%), and KHI 55 (91.8 %). The results showed that haploid inducers with the highest HIR were from the moderate group while haploid inducers with the highest ISR were noticed from each group. In this growing season, there was no clear association between HIR and ISR where haploid inducers with high haploid production tend to possess moderate inducer seed set.

In the rainy season, the highest HIR was found in three haploid inducers: KHI 42 (1.8%), KHI 50 (1.8%), and KHI 66 (1.5%). In addition, four haploid inducers, KHI 56 (97.5%), KHI 5 (95.6%), KHI 42 (95.2%), and KHI 72 (94.3%), had the highest ISR. In this season, both HIR and ISR seemed to be correlated as more haploids were induced from the inducer genotypes with higher ISR. For instance, KHI 42 having 95.2% of inducer seed set showed a 1.8% haploid frequency per cross.

## 3. Materials and Methods

### 3.1. Plant Materials

A total of 14 Stock-6-derived maize inducer lines were used in this study (Table 4). The origin and pedigree of these lines were described by Dermail et al. [16]. Briefly, base populations were established from intercrossing between temperate inducer Stock 6 with 2.3% of HIR [7] and eight tropical corn genotypes including three waxy corn (KND, TL, and TB), two sweet corn (101L and WST), and three field corn genotypes (Pacific339, NS3, and NSX). 

About 78 S3 families were obtained in the dry season of 2019, and those materials were further utilized for dual studies, namely, the first study on selection gain through the modified ear-to-row method [16] and the second study on seasonal variation which was emphasized in this paper. Unweighted selection index, a simultaneous selection method without considering certain economic values of traits of interest, [20] was performed at the preliminary study to sort all 78 genotypes based on HIR, inducer seed rate, and agronomic score. The top rank genotype had the highest indices (z). Then, we determined the thresholds of selection indices (z) to categorize the sorted genotypes into three groups: high (genotypes with z ≥ 2.5), moderate (genotypes with 0.5< z < 2.5), and low (genotypes with z ≤ 0.5). According to the groups established, representative five, five, and four genotypes, respectively, were selected based on the top rank within the group. The list of the genotypes is presented in Table 4.

### 3.2. Field Experiment

Fourteen Stock-6-derived maize inducer lines were evaluated under field conditions at the Agronomy Field Crop Research Station, Khon Kaen University, Khon Kaen, Thailand (16°28’27.7” N, 102°48’36.5” E; 190 m above sea level (masl)) in two different growing seasons, namely, the dry season (November 2020–February 2021) and the rainy season (April–August 2021). The inducer lines were assigned in a randomized complete block design (RCBD) with three replications. The plot size was 4 rows of 5 m in length. The plant spacing was 0.75 m × 0.25 m; thus, the plant density was about 80 plants per plot. In both seasons, standard agronomic practices were used following the Department of Agriculture, Thailand, recommendations (Thai Agricultural Practice, Department of Agriculture, Thailand. Available online: http://www.doa.go.th (accessed on 12 June 2022)) including land preparation, fertilization, irrigation, and pest, disease, and weed control.

The weather profiles such as daily minimum and maximum temperatures (°C), relative humidity (%), precipitation rate (mm), and solar radiation (MJ m^−2^ day^−1^) were collected daily during the experiment from the Agricultural Weather Station, Agronomy Field Research Station, Khon Kaen University, Thailand. Growing degree days (GDD) [21] of each growing season were calculated for every day after planting by accumulating the temperature per day using the following formula:

°C day−1 = ((Tmin + Tmax)/2) – 10(1)

where Tmin is the daily minimum temperature (°C) and Tmax is the daily maximum temperature (°C). The base temperature for maize is 10 °C.

### 3.3. Haploid Induction and Ploidy Identification 

Two commercial F_1_ hybrids S7328 and P789 released by Syngenta Seeds and Pacific Seeds, respectively, were used as the donor parent or tester. Three staggered planting dates for donor genotypes with intervals of seven days were carried out to ensure flowering synchrony. The donor was placed adjacent to the inducer plots. About ten inducer plants per plot were selected and self-pollinated for inducer maintenance, and those selected plants per plot were also individually cross-pollinated to ten ears of each donor genotype for haploid induction.

All dried seeds from each donor ear were classified based on the *R1-nj* anthocyanin biomarker on the crown (top endosperm tissue) and scutellum of the embryo [22]. They were classified into putative diploid if the seeds showed purple colorations of the endosperm and embryo and putative haploid if the seeds showed purple endosperm and colorless embryo. Haploid induction rate (HIR) and inducer seed rate (ISR) were calculated as follows:

(2) $$\mathrm{HIR}\;(\%)=\frac{\mathrm{seed}\;\mathrm{number}\;\mathrm{of}\;\mathrm{putative}\;\mathrm{haploid}}{\mathrm{total}\;\mathrm{seed}\;\mathrm{number}\;\mathrm{per}\;\mathrm{ear}}\text{ × }100$$

(3) $$\mathrm{ISR}\;(\%)=\frac{\mathrm{seed}\;\mathrm{number}\;\mathrm{of}\;\mathrm{inducer}}{\mathrm{total}\;\mathrm{seed}\;\mathrm{number}\;\mathrm{per}\;\mathrm{ear}}\text{ × }100$$

### 3.4. Data Collection

Germination rate was calculated as the percentage of emerged seedlings per plot at 14 days after sowing (DAS). At the reproductive stage, the following agronomic traits were recorded using the sample basis of 10 plants per plot except for flowering dates which were plot-based measurements: plant height (cm), as a distance from ground level to the node bearing the flag leaf; ear height (cm), as a distance from ground level to the node bearing the uppermost ear; days to anthesis, as the number of days from sowing to when 50% of the plants shed pollen; days to silking, as the number of days from sowing until silks emerged on 50% of the plants; pollen-shed duration (days), as the number of days from the last day of pollen shedding minus the first day of pollen shedding; primary tassel branching, as the number of primary tassel branches having anthers emerged; total tassel branching, as the total number of tassel branches including the primary and secondary branches; tassel length (cm), as the length from the lowermost primary branch to the tip of the tassel; and spike length (cm), as the length from the uppermost branch to the tip of the spike.

Yield components were measured on ten ears per plot at the physiological maturity stage approximately 45 days after pollination (DAP). The parameters included ear weight (g), ear diameter (cm), ear length (cm), husk cover, the number of rows per ear, the number of kernels per row, cob diameter (cm), and kernel diameter (cm). 

### 3.5. Statistical Analysis

All observed data were subjected to Bartlett’s test for homogeneity of variance and the Shapiro–Wilk test for normality. Then, combined analysis of variance was performed considering season and genotype as random effects and replication as a fixed effect by using PROC MIXED of SAS ver. 9.0 (SAS Institute. SAS for Windows Version 9.0; SAS Institute: Cary, NC, United States, 2002) using the following linear model:

Y_ijk_ = µ + s_i_ + r_j_(s_i_) + g_k_ + s_i_g_k_ + ε_ijk_ (4)

where i = 1, 2; j = 1, 2, 3; k = 1, 2, 3…14; Y_ijk_ denotes the phenotype of genotype k in season i and replication j; µ is the overall mean; s_i_ is the effect of season i; r_j_(s_i_) is the effect of replication k nested within season i; g_k_ is the effect of genotype k; s_i_g_k_ is the effect of the interaction between season i and genotype k; and ε_ijk_ is the pooled error of season i, replication j, and genotype k.

Duncan’s multiple range test (DMRT) at 0.05 probability level was used for mean comparison [23].

## 4. Discussion

### 4.1. Phenotype of Maize Haploid inducers Is Affected by Genotype, Season, and their Interaction

In this study, all agronomic traits and haploid induction rate clearly showed quantitative inheritance, with continuous variation, and respective phenotypes resulting from genotype, environment, and the interaction between genotype and environment [24]. The significance of genotype for all observed traits may be explained by the fact that the 14 haploid inducers used in this study were derived from different groups, based on haploid induction rate, R1-nj expression, and agronomic trait performance. Genotype had a strong impact on for ISR and several agronomic traits because we included several tropical non-inducer genotypes as founder parents, which had different plant ideotypes including plant stature, flowering behaviors, and yields, and the alleles of those traits may be segregated during population improvements. ISR was defined as the proportion of inducer seed per ear that expressed the R1-nj anthocyanin marker. Chaikam et al. [25] reported that tropical maize showed a high frequency of complete inhibition of R1-nj due to the presence of C1-I, a dominant anthocyanin inhibitor gene. In addition, the minor contribution of genotype on HIR might be due to the common founder parent used which was Stock 6.

The significant effect of season on most attributes indicated the presence of weather dynamics of tropical savanna climate from the dry season to the rainy season which altered the phenotypic means of haploid inducers. This result corroborated previous studies where seasonal variation under tropical savanna regions significantly contributed to the agronomic traits and yield components of vegetable corn [26,27]. Meanwhile, the predominant effect of GSI on HIR implied that the HIR of haploid inducers was sensitive to seasonal variation and each genotype responded differently in different growing seasons. GSI reflects genotype by environment interaction (GEI) and illustrates the failure of genotypes to be stable in different environmental conditions [28].

### 4.2. Seasonal Variation Is Responsible for Unstable Performance of Maize Haploid Inducers 

In our region, the tropical savanna climate has two common maize-growing seasons, namely, the rainy and the dry seasons. In this study, the rainy season was identified with a high precipitation rate, relative humidity, and daily temperature, while the opposite weather features were clearly noticed in the dry season. The effect of contrasting temperature profiles over two seasons on maize phenology was illustrated with growing degree days [21,29]. Growing degree days (GDD) is one of the thermal units and is represented by the accumulated temperature above a base level for a whole crop growing period [30]. The values vary depending on the environmental conditions [31]. In maize, it has been adopted for estimating plant phenology including flowering, maturity, and harvest dates [32], for assessing the suitable growing environments [33], and for simulating crop growth such as in hybrid-maize models [34]. Our study found that the phenology of overall haploid inducer lines grown in the rainy season was faster than in the dry season, as indicated by higher GDDs but shorter days to anthesis and maturity. This corroborated previous studies in maize reporting that a warm-temperature zone had a higher accumulation of GDD and shorter sowing–silking interval than a cold-temperature zone in the tropical climates of Thailand and Mexico [35], the semi-humid and semi-arid climates of China [33], the semi-arid and humid subtropical of Texas [36], and the temperate climate of Czech Republic [37]. Lower daily temperatures led to increased maize vegetative growth [31] while, on the contrary, warmer daily temperatures accelerated the maize growth rate, which in turn shortened both vegetative and reproductive stages [38]. In our study, we also noticed variations during the vegetative period but fewer variations during the reproductive period. The result confirmed previously similar studies in maize, stating that the silking–maturity period was less variable than the sowing–silking period [39,40].

Weather plays critical roles in crop growth and phenology [41,42], aboveground biomass, and yield [31]. Among weather parameters, temperature, solar radiation, and precipitation are three major factors determining the development rate, biomass, yield components, and grain yield of maize under diverse climate regions from subtropical to temperate [31,37,43,44]. Our findings suggest that the dry season is more suitable than the rainy season for growing maize haploid inducers, as indicated by taller plant stature and ear position, later flowering, bigger tassel size, higher tassel branching, longer pollen-shed duration, larger ear and seed sizes, better seed set, and higher haploid induction rate. Kebede et al. [15] reported that the winter season was more suitable for haploid induction than the summer season in the subtropical climate of Mexico. Colder temperatures resulted in the maize plants being left for longer durations in the field [32]. Since the dry matter accumulation in maize relied on plant growth rate and growth duration [40], a prolonged vegetative stage and photoperiod made the maize utilize the abundant resources including radiation, water, and nutrients [45] to produce more photosynthates for more leaves [46,47], taller plant stature [36], later flowering and harvesting times without significantly altering the pollen production [48,49], and higher aboveground biomass [50] and grain yield [51]. In addition, cool nights in the dry season may benefit maize plants especially at the flowering and grain-filling stages by enhancing the accumulation of assimilates through photosynthesis and respiration processes [49,52].

In contrast, the conditions during the rainy season were unfavorable for haploid induction due to early flowering, smaller tassel size, and short pollen-shed duration, and prevalence of tropical diseases such as downy mildew, northern corn leaf blight, and rust. This led to poor pollination and seed set of both inducer and test crosses. High daily temperature, relative humidity, and precipitation during the rainy season were likely responsible. De La Fuente et al. [2] noticed that high precipitation rates during the pollination period dropped the haploid induction rate due to the significant damage by fungal ear diseases. In maize, increasing temperature and GDD significantly shorten the growth period and rate, resulting in reduced post-silking aboveground biomass [31], 1000-kernel weight, and grain yield [53]. There was a negative association between increasing temperature and maize yield [54,55] in which each additional GDD30^+^ led to a yield reduction of about 1.0–1.2% [29,56]. High temperatures contributed to yield reduction, not only because of the damage to maize flowers during daytime [30], but also because of increased respiration and decreased net dry matter accumulation during nighttime [57]. The impairment of maize flowers was further explained by the pollen sterilility [38,58] and the reduced floret elongation rate and number [59,60], leading to poor fertilization synchronization and high kernel abortion [61]. Since the potential kernel number and kernel weight rely on the number of florets [62] and the kernel growth rate [44], respectively, this condition may cause a reduction in the final seed set as the maize grain yield is composed of the kernel number per unit area and weight per kernel [63]. In addition, nitrogen is one of essential macronutrients for optimum maize growth, photosynthesis, and grain formation [64]; thus, excessive precipitation combined with warm daily temperatures during the rainy season may contribute to more yield losses due to nitrogen leaching and denitrification [65,66,67]. 

### 4.3. Crossover Performance of Maize Haploid Inducers and Implication for Haploid Induction 

The significant interaction between genotype and season was revealed by the inconsistent performance of haploid inducers to produce haploid seeds over contrasting maize growing seasons. When cool temperatures during daytime and nighttime and low relative humidity occurred during the dry season, two haploid inducers, KHI 65 and KHI 66, were the best option. In contrast, when the weather conditions were getting worse, indicated by increasing temperatures, rainfall, and relative humidity, other haploid inducers such as KHI 42 and KHI 50 were the most viable option. In in vivo haploid induction, flowering synchrony between the silking of donor germplasm and the anthesis of haploid inducers is important, and occasionally incompatible pollinations occur when it comes to the male reproductive organ which has a shorter flowering period than the female one. In general, five to seven days are the total pollen-shed duration of maize inbred lines [49,68], while the stigmas of female plants are still receptive for 13 days after the silking initiation [69]. Since the pollen-shed duration of our haploid inducers was not only shorter than that normally reported, but also affected by the seasonal variation from five days in the dry season to three days in the rainy season, performing multiple planting dates with constant interval days of haploid inducers as the pollinator was feasible to extend the pollen production for haploid induction. The situation might be more complicated if vast germplasm with diverse silking dates are included. If so, utilizing two or more haploid inducers, having similar HIR with a different anthesis date, and multiple planting dates for those inducers with irregular interval days can be applied. 

## 5. Conclusions

The effects of season, genotype, and genotype by season were significant for all traits except that season factored on inducer seed rate. Under a tropical savanna climate, the dry season was more suitable for growing haploid inducers for haploid production and inducer maintenance, as they revealed better agronomic performance and seed set, delayed flowering dates, and higher haploid induction rate (HIR). Two haploid inducers KHI 65 and KHI 66 (HIR~3.3–3.5%) were proposed to be used in the dry season when the weather conditions were suitable for maize growth, flowering, and seed development. Meanwhile, the other two inducers, KHI 42 and KHI 50 (HIR~1.8%), were the best option in the rainy season when high temperature, high precipitation, relative humidity, and disease incidence occurred. Applying two or more haploid inducers and using multiple planting dates for those inducers was suggested to achieve optimal seed set and potential haploid seeds per induction cross.

## Figures and Tables

**Table 1:** Mean squares from the combined ANOVA on HIR, ISR, yield components, and agronomic traits across two seasons between 2020 and 2021.

**SOV**	**df**	**HIR**	**ISR**	**Yield Components**							
				**EW**	**EL**	**ED**	**HC**	**NRE**	**NKR**	**CD**	**KD**
Season (S)	1	10.1 **	164.7 ns	57,150.1 **	76.9 **	40.3 **	6.3 **	403.3 **	1931.5 **	10.9 **	1.3 **
		(14.1)	(0.5)	(79.5)	(13.3)	(60.4)	(10.4)	(47.4)	(54.1)	(47.7)	(46.5)
Rep/S (a)	4	0.1	41.9	3.7	0.3	0.0	0.1	0.4	1.9	0.0	0.0
		(0.7)	(0.5)	(0.0)	(0.2)	(0.2)	(0.7)	(0.2)	(0.2)	(0.2)	(0.9)
Genotype (G)	13	1.8 **	1674.0 **	856.0 **	20.9 **	1.0 **	2.8 **	14.1**	87.2 **	0.5 **	0.1 **
		(33.0)	(64.4)	(15.5)	(47.0)	(17.9)	(59.8)	(21.5)	(31.7)	(26.6)	(28.0)
G × S	13	2.3 **	767.9 **	171.6 **	14.8 **	1.0 **	1.0 **	16.9 **	25.7 **	0.4 **	0.1 **
		(41.5)	(29.5)	(3.1)	(33.4)	(18.7)	(22.6)	(25.8)	(9.4)	(22.1)	(21.8)
Pooled error (b)	52	0.1	33.3	25.9	0.7	0.0	0.1	0.8	3.2	0.0	0.0
		(10.7)	(5.1)	(1.9)	(6.1)	(2.8)	(6.6)	(5.2)	(4.6)	(3.3)	(2.8)
C.V.(a)(%)		29.0	8.4	4.2	5.4	5.9	14.9	5.9	8.3	6.0	12.4
C.V.(b)(%)		30.8	7.5	11.3	8.7	6.3	13.2	8.5	10.7	6.3	6.2
**SOV**	**df**	**Agronomic traits**									
		**GR**	**PSD**	**DTA**	**DSI**	**PH**	**EH**	**PTB**	**TTB**	**TSL**	**SPL**
Season (S)	1	342.5 *	19.6 **	1288.6 **	3375.7 **	29,385.2 **	10,906.4 **	82.4 **	199.3 **	2979.6 **	1818.3 **
		(6.3)	(41.8)	(57.7)	(78.8)	(42.4)	(35.7)	(14.9)	(20.4)	(72.3)	(69.1)
Rep/S (a)	4	24.7	0.1	2.9	1.5	98.7	41.9	0.2	3.4	4.2	5.9
		(1.8)	(0.9)	(0.5)	(0.1)	(0.6)	(0.5)	(0.1)	(1.4)	(0.4)	(0.9)
Genotype (G)	13	213.9 **	0.7 **	44.6 **	46.8 **	2369.2 **	1234.6 **	24.6 **	42.8 **	51.0 **	29.3 **
		(51.4)	(19.2)	(26.0)	(14.2)	(44.5)	(52.5)	(57.6)	(57.0)	(16.1)	(14.5)
G × S	13	72.0 **	0.9 **	11.7 **	9.3 **	400.9 **	135.5 **	8.6 **	11.3 **	24.1 **	22.8 **
		(17.3)	(24.2)	(6.8)	(2.8)	(7.5)	(5.8)	(20.2)	(15.0)	(7.6)	(11.3)
Pooled error (b)	52	24.1	0.1	3.8	3.3	66.9	32.5	0.8	1.2	2.9	2.2
		(23.2)	(13.8)	(8.9)	(4.0)	(5.0)	(5.5)	(7.2)	(6.1)	(3.7)	(4.3)
C.V.(a)(%)		5.8	8.3	3.0	2.2	7.3	9.8	4.0	14.0	7.6	14.0
C.V.(b)(%)		5.8	8.9	3.4	3.2	6.0	8.6	8.2	8.1	6.3	8.5

HIR, haploid induction rate; ISR, inducer seed rate; EW, ear weight; EL, ear length; ED, ear diameter; HC, husk cover; NRE, the number of rows per ear; NKR, the number of kernels per row; CD, cob diameter; SD, seed diameter; GR, germination rate; PSD, pollen-shed duration; DTA, days to anthesis; DSI, days to silking; PH, plant height; EH, ear height; PTB, primary tassel branch; TTB, total tassel branch; TSL, tassel length; SPL, spike length; ns, not significant; and *,**, significant at *p* ≤ 0.05 and *p* ≤ 0.01, respectively. The number within the parentheses is the percentage of sum squares to the total sum of squares.

**Table 2:** Means of agronomic traits, yield components, and haploid induction ability in maize haploid inducers in the dry and rainy seasons.

**Season**	**Agronomic Traits**									
	**GR** **(%)**	**PSD** **(d)**	**DTA** **(d)**	**DSI** **(d)**	**PH** **(cm)**	**EH** **(cm)**	**PTB**	**TTB**	**TSL** **(cm)**	**SPL** **(cm)**
Dry	83.3 ± 1.2	4.5 ± 0.1	61.1 ± 0.5	63.9 ± 0.6	154.1 ± 4.0	77.7 ± 2.7	11.7 ± 0.4	14.8 ± 0.5	33.0 ± 0.6	22.1 ± 0.5
Rainy	87.4 ± 1.3	3.5 ± 0.1	53.3 ± 0.5	51.2 ± 0.5	116.7 ± 2.7	54.9 ± 2.1	9.7 ± 0.4	11.7 ± 0.4	21.1 ± 0.6	12.8 ± 0.5
*p*-value	0.033	<0.001	<0.001	<0.001	<0.001	<0.001	0.001	0.004	<0.001	<0.001
**Season**	**HIR** **(%)**	**ISR** **(%)**	**Yield Components**							
			**EW** **(g)**	**EL** **(cm)**	**ED** **(cm)**	**HC**	**NRE**	**NKR**	**CD** **(cm)**	**KD** **(cm)**
Dry	1.6 ± 0.2	78.7 ± 1.9	71.1 ± 2.4	10.4 ± 0.2	3.7 ± 0.1	2.4 ± 0.1	13.0 ± 0.2	21.5 ± 0.5	2.3 ± 0.1	0.8 ± 0.1
Rainy	0.9 ± 0.2	75.9 ± 4.0	19.0 ± 1.7	8.5 ± 0.5	2.3 ± 0.1	1.8 ± 0.1	8.6 ± 0.5	11.9 ± 0.8	1.6 ± 0.1	0.5 ± 0.1
*p*-value	0.008	0.562	<0.001	0.003	<0.001	0.007	<0.001	<0.001	<0.001	<0.001

Values are means ± SE. GR, germination rate; PSD, pollen-shed duration; DTA, days to anthesis; DSI, days to silking; PH, plant height; EH, ear height; PTB, primary tassel branch; TTB, total tassel branch; TSL, tassel length; SPL, spike length; HIR, haploid induction rate; ISR, inducer seed rate; EW, ear weight; EL, ear length; ED, ear diameter; HC, husk cover; NRE, the number of rows per ear; NKR, the number of kernels per row; CD, cob diameter; and KD, kernel diameter.

**Table 3:** Means of 14 haploid inducers on haploid induction rate (HIR) and inducer seed rate (ISR) evaluated in the dry season of 2020/2021 and the rainy season of 2021.

Group	Haploid Inducers	Dry Season		Rainy Season	
		HIR (%)	ISR (%)	HIR (%)	ISR (%)
High	KHI 49	1.8 cd (A)	74.0 bc (A)	0.0 e (B)	82.9 b (A)
	KHI 59	0.8 ef (A)	81.3 b (A)	0.3 e (B)	88.7 ab (A)
	KHI 54	2.5 bc (A)	62.5 de (B)	0.9 d (B)	72.8 c (A)
	KHI 42	0.8 ef (B)	93.6 a (A)	1.8 a (A)	95.2 a (A)
	KHI 47	1.7 cd (A)	94.2 a (A)	1.6 ab (A)	92.1 ab (A)
Moderate	KHI 5	1.8 cd (A)	92.0 a (A)	0.0 e (B)	95.6 a (A)
	KHI 65	3.4 a (A)	71.4 cd (A)	0.0 e (B)	66.4 cd (A)
	KHI 66	3.3 ab (A)	75.2 bc (A)	1.5 ab (B)	53.5 e (B)
	KHI 50	1.1 def (B)	81.0 bc (A)	1.8 a (A)	61.0 de (B)
	KHI 80	0.6 f (A)	63.8 de (A)	0.0 e (B)	0.0 f (B)
Low	KHI 56	1.0 def (B)	79.4 bc (B)	1.5 ab (A)	97.5 a (A)
	KHI 61	0.4 f (B)	80.9 bc (A)	0.9 d (A)	71.9 c (A)
	KHI 72	1.5 de (A)	60.7 e (B)	1.3 bc (A)	94.3 a (A)
	KHI 55	1.7 d (A)	91.8 a (A)	1.0 cd (B)	90.6 ab (A)

Means with different letters outside parenthesis in the same column are significantly different by Duncan’s multiple range test (DMRT) at 0.05 probability level. Means with different letters within parenthesis in the same row and trait are significantly different by Tukey’s honest significant difference test (HSD) at 0.05 probability level.

**Table 4:** Brief profiles of fourteen Stock-6-derived inducer lines used in this study.

No.	Genotypes	Pedigree	Group ^1^
1	KHI 49	WST/Stock6-S(C6)-IDLT2A-28-B	High
2	KHI 59	WST/Stock6-S(C6)-IDLT2A-WS-B	High
3	KHI 54	WST/Stock6-S(C6)-IDLT2A-34-1-B	High
4	KHI 42	TL/Stock6-S(C6)-IDLT1B-93-B	High
5	KHI 47	WST/Stock6-S(C6)-IDLT2A-24-B	High
6	KHI 5	NSX/Stock6-S(C6)-IDLT1A-110-B	Moderate
7	KHI 65	KND/Stock6-S(C6)-IDLT2B-22-B	Moderate
8	KHI 66	TB/Stock6-S(C6)-IDLT3-4-B	Moderate
9	KHI 50	WST/Stock6-S(C6)-IDLT2A-29-B	Moderate
10	KHI 80	TB/Stock6-S(C6)-IDLT4-4-B	Moderate
11	KHI 56	WST/Stock6-S(C6)-IDLT2A-36-B	Low
12	KHI 61	KND/Stock6-S(C6)-IDLT2B-15-B	Low
13	KHI 72	TB/Stock6-S(C6)-IDLT4-24-B	Low
14	KHI 55	WST/Stock6-S(C6)-IDLT2A-35-B	Low

^1^ Based on unweighted selection index (z) of three attributes, namely, HIR, inducer seed rate, and agronomic score. High: genotypes with z ≥ 2.5; moderate: genotypes with 0.5 < z < 2.5; and low: genotypes with z ≤ 0.5.

## Data Availability

The data that support the findings of this study are available from the corresponding author upon reasonable request.
